# Phosphate-Solubilizing *Pseudomonas* sp. Strain WS32 Rhizosphere Colonization-Induced Expression Changes in Wheat Roots

**DOI:** 10.3389/fmicb.2022.927889

**Published:** 2022-06-30

**Authors:** Kangmiao Ou, Xiangyi He, Ke Cai, Weirong Zhao, Xiaoxun Jiang, Wenfeng Ai, Yue Ding, Yuanyuan Cao

**Affiliations:** ^1^School of Life Sciences, Anhui Agricultural University, Hefei, China; ^2^Anhui Province Key Laboratory of Farmland Ecological Conservation and Pollution Prevention, Anhui Agricultural University, Hefei, China

**Keywords:** *Pseudomonas* sp. strain WS32, wheat, colonization, growth promotion, phosphorus nutrient, RNA-seq technology, differential gene expression

## Abstract

Rhizosphere colonization is a pre-requisite for the favorable application of plant growth-promoting rhizobacteria (PGPR). Exchange and mutual recognition of signaling molecules occur frequently between plants and microbes. Here, the luciferase *lux*AB gene was electrotransformed into the phosphate-solubilizing strain *Pseudomonas* sp. WS32, a type of plant growth-promoting rhizobacterium with specific affinity for wheat. A labeled WS32 strain (WS32-L) was applied to determine the temporal and spatial traits of colonization within the wheat rhizosphere using rhizoboxes experimentation under natural condition. The effects of colonization on wheat root development and seedling growth were evaluated, and RNA sequencing (RNA-seq) was performed to explore the transcriptional changes that occur in wheat roots under WS32 colonization. The results showed that WS32-L could survive in the wheat rhizosphere for long periods and could expand into new zones following wheat root extension. Significant increases in seedling fresh and dry weight, root fresh and dry weight, root surface area, number of root tips, and phosphorus accumulation in the wheat leaves occurred in response to WS32 rhizosphere colonization. RNA-seq analysis showed that a total of 1485 genes in wheat roots were differentially expressed between the inoculated conditions and the uninoculated conditions. Most of the transcriptional changes occurred for genes annotated to the following functional categories: “phosphorus and other nutrient transport,” “hormone metabolism and organic acid secretion,” “flavonoid signal recognition,” “membrane transport,” and “transcription factor regulation.” These results are therefore valuable to future studies focused on the molecular mechanisms underlying the growth-promoting activities of PGPR on their host plants.

## Introduction

The rhizosphere, which is referred to as the small region of soil directly surrounded and affected by the plant root system (Grover et al., 2021), is an active region of microbial activities and is considered to be one of the most complex natural ecosystems (Mendes et al., 2013). In the rhizosphere, microorganisms and plants interact, which can lead to changes in morphology, growth, metabolism, and development; these interactions ultimately maintain the stability of the plant rhizospheric ecosystem and ensure proper plant nutrition and growth (Artursson et al., 2006; Tak et al., 2013).

Bacteria that are present in the rhizosphere, that promote plant growth and nutrient absorption and that help prevent infection of plant pathogens, are designated plant growth-promoting rhizobacteria (PGPR) (Puri et al., 2016; Chandrasekaran et al., 2019). The mechanisms by which PGPR promote plant growth include solubilizing phosphorus; dissolving potassium; fixing nitrogen; producing indoleacetic acid, siderophores, and antimicrobial substances; and triggering induced systemic resistance (Idris et al., 2007; Zaidi et al., 2009; Baig et al., 2012; Mohite, 2013; Yin et al., 2015; Cordovez et al., 2019).

However, the efficacy of PGPR to play a growth-promoting role can decrease due to a low capacity to colonize rhizosphere. Effective colonization of PGPR strains involves mutual recognition between plants and microbes. Plant lectins play a vital role in the mutual recognition process between plants and microorganisms. Lectins are a class of proteins that are secreted by plants and can specifically and non-enzymatically bind to carbohydrate groups on the surface of bacteria, agglutinate specific bacteria at the plant roots and then initiate a reaction that leads to rhizosphere colonization by bacteria (Komath et al., 2006; Liu et al., 2019). Studying the survival and colonization of PGPR in the rhizosphere requires the use of methods that can easily track the target strains in a soil environment in which there is a large number of indigenous microorganisms (Prosser, 1994). Owing to its convenience and sensitivity, *lux*AB fluorescence labeling technology, which is a molecular marker technology, has been widely used to trace target microorganisms in complex environments (Tang et al., 2004; Zhu et al., 2013; Liu et al., 2019). Colonies of *lux*AB-tagged strains can be observed in the dark with just the naked eye because they adequately fluoresce and are easily countable by the dilution plate counting method (Prosser, 1994), even when they are vastly outnumbered by colonies of indigenous microorganisms (Liu et al., 2019).

Plant root exudates influence the distribution and composition of rhizosphere microorganisms. Moreover, the activities of rhizosphere microorganisms affect the nutrient absorption and growth of plants (Liu et al., 2019). However, the molecular mechanisms underlying the interactions between plants and microorganisms are still not entirely understood. Recently, rapid advances in RNA sequencing (RNA-seq) and associated bioinformatics tools have led to the creation of revolutionary tools for studies on the mechanisms of plant-bacterium interactions. Cervantes-Gámez et al. (2016) showed by RNA-seq analysis that arbuscular mycorrhizal fungal symbiosis can induce transcriptional changes in *Solanum lycopersicum* leaves. Using RNA-seq analysis, Yuan et al. (2016) revealed differential gene expression in response to different Rhizobium strains in soybean roots. Kawahara et al. (2012) utilized RNA-seq analysis to explore the mechanisms underlying the interactions between rice and rice blast fungi. Most related studies in this field have focused on revealing relationships between bacteria that have a symbiotic or parasitic relationship with their host plants, but little attention has been given to exploring the mechanisms underlying plant growth-promoting rhizobacterium-plant interactions.

We previously isolated a phosphate-solubilizing strain, *Pseudomonas* sp. strain WS32, which is a type of plant growth-promoting rhizobacterium with affinity for wheat (Zhang J. et al., 2012). Here, a pTR102 plasmid harboring the luciferase *lux*AB gene was introduced into WS32 *via* electrotransformation. The WS32 strain was labeled (WS32-L) and used to investigate the colonization dynamics of the strain in a wheat rhizosphere using rhizoboxes experimentation under natural condition. The effects of colonization on wheat root development and seedling growth were evaluated. Moreover, RNA-seq transcriptional profiling of wheat roots colonized by phosphate-solubilizing *Pseudomonas* sp. strain WS32 was performed to better understand the molecular mechanisms of host plant responses induced by PGPR rhizosphere colonization. These results may be useful for further characterization of the relationships between PGPR and their host plants.

## Materials and Methods

### Plant Materials, Strains, Media, and Soil

Wheat (*Triticum aestivum* W52) seeds were obtained from the School of Agronomy, Anhui Agricultural University, China. The phosphate-solubilizing *Pseudomonas* sp. strain WS32 was isolated from the rhizosphere of wheat plants by members of our laboratory (Zhang J. et al., 2012). The plant growth-promoting traits of WS32 include the solubilization of phosphate (at a rate of 31.69 μg⋅mL^–1^) and high production of siderophores (+++++) (Zhang J. et al., 2012). *Escherichia coli* strain WA803 harboring the plasmid pTR102:*lux*AB, *km*, *tet*, was supplied by the College of Life Sciences, Nanjing Agricultural University, China.

We used Luria-Bertani (LB) media that comprised the following components (g⋅L^–1^): bacto-tryptone (10.0), yeast extract (5.0), and NaCl (10.0) (pH 7.0). The Super Optimal broth with Catabolite repression (SOC) media comprised the following components (g⋅L^–1^): peptone (20.0), yeast extract (5.0), NaCl (0.5), KCl (0.186), MgCl_2_⋅6H_2_O (0.95), MgSO_4_⋅7H_2_O (1.2), and glucose (3.6) (pH 7.0).

Wheat seedlings were grown in unsterilized yellow cinnamon soil. The available phosphorus, available potassium, and ammonium nitrogen levels were 15.37, 152.36, and 16.35 mg⋅kg^–1^ soil, respectively.

### Electrotransformation of *Pseudomonas* sp. Strain WS32

pTR102 plasmids were extracted from cells of *E. coli* WA803 (cultured overnight) using a SanPrep Column Plasmid DNA Miniprep Kit (Sangon Biotech Co., Ltd., Shanghai, China). Moreover, competent *Pseudomonas* sp. strain WS32 cells were obtained for electrotransformation as follows. The cells were cultured in LB media at 28°C until the optical density at 600 nm reached 0.6, collected *via* centrifugation (8000 r⋅min^–1^, 5 min, 4°C), washed with pre-chilled ultrapure water, and then resuspended in 40 μL of ice-cold 10% (v/v) glycerol. Then, a mixture of 40 μL of competent cells and 2 μL of plasmids was electroporated at 2.5 kV, 25 μF, and 200 Ω by an ECM830 electroporation unit (Harvard Apparatus, Holliston, MA, United States). The sample was subsequently resuspended in 1 mL of SOC media and incubated at 28°C for 1.5 h at 160 r⋅min^–1^. The sample was then spread onto plates containing solid LB media supplemented with 50 μg⋅mL^–1^ kanamycin and 50 μg⋅mL^–1^ tetracycline. After 3 days of incubation at 28°C, 10% capraldehyde was added in a dropwise manner to the inner surfaces of the cover of these plates in the dark. Transformants were selected based on their fluorescence, and then verified by 16S rRNA gene sequencing. Cells of the transformant were plated successively onto solid LB media that lacked antibiotics. The fluorescence activity in the dark and the kanamycin and tetracycline resistance of the colonies were subsequently measured to verify the genetic stability of the transformants. The standard growth curves (in LB media), and plant growth-promoting traits, including phosphate solubilization and siderophore production, of strain WS32 and the *lux*AB-labeled strain WS32-L were compared. Phosphate solubilization was evaluated using the phosphomolybdate-blue-based colorimetric method (Gull et al., 2004), and siderophore production was measured using a chrome azurol S assay (Liu et al., 2014).

### Colonization of the Wheat Rhizosphere by Strain WS32-L

Wheat seeds were immersed in water for 4 h, and then, those floating on the surface of water were removed. After disinfection with 75% ethanol for 5 min and four washes with sterilized distilled water, the seeds were transferred to a sterilized culture dish containing sterilized moistened filter paper and maintained at 25°C for 2–3 days for germination. Moreover, WS32-L cells were cultured in LB media at 28°C and 160 r⋅min^–1^ until the OD_600_ of the culture reached 0.8. The germinated wheat seeds were then immersed in the WS32-L culture for 30 min and then sown in rhizoboxes (one seed per rhizobox; 13 cm long, 1 cm wide, 25 cm deep) that contained unsterilized yellow cinnamon soil. The germinated wheat seeds that were immersed in LB media for 30 min were sown under the same conditions used as the controls. The germinated wheat plants were grown outdoors under natural conditions (average rainfall = 111.6 mm and temperature range 18–31°C). The wheat roots were sampled on days 6, 12, and 36 to assess the colonization dynamics of the WS32-L strain. For sampling, soil particles were shaken off the wheat roots carefully. The roots were then cut into 2 cm long segments *via* sterile scissors from top to bottom. Each segment was put into a triangular flask containing 100 mL of sterilized physiological saline solution and glass beads. After rotated (160 r⋅min^–1^) at 28°C for 1 h, the samples were vortexed vigorously for 2 min to release the attached cells. Serial dilutions were performed, and 100 μL of each dilution was spread onto solid LB media supplemented with 50 μg mL^–1^ kanamycin and 50 μg⋅mL^–1^ tetracycline. After incubation for 3 days at 28°C, the number of bacteria were determined by counting the fluorescent colonies.

### Wheat Plant Development and Measurements of Phosphorus Contents in the Leaves and Rhizosphere Soil

Germinated wheat seeds were immersed in the WS32 culture solution for 30 min and then sown into rhizoboxes containing unsterilized yellow cinnamon soil using the same methods and conditions as those used for the colonization experiment. The wheat seedlings were sampled 25 days later. The total root length (TRL), root surface area (RSA), root average diameter (RAD), root volume (RV), and number of root tips and forks were measured by a WinRHIZO scanner (Regent Instruments, Inc., Quebec, Canada). Moreover, the seedling fresh weight and root fresh weight were measured, and the seedling dry weight and root dry weight were also measured after the wheat plant samples were dried at 60°C and reached a constant weight. The phosphorus content in the wheat leaves was determined by the vanadium-molybdenum blue method (Murphy and Riley, 1962), and the available phosphorus in the soil was determined by the Bray method (Bray and Kutrz, 1945). All the treatments were replicated three times. Root samples were collected, frozen quickly in liquid nitrogen and then stored at −80°C for RNA-seq.

### Ribonucleic Acid Library Construction and Sequencing

Total RNA from the roots (three biological replicates) of inoculated and uninoculated wheat was extracted using an RNAiso plus kit (TaKaRa, Dalian, China) according to the manufacturer’s protocol. The RNA quality was assessed *via* electrophoresis of 1% agarose gels. The concentration, as well as the A260/280 and A260/230 ratios of RNA, were measured by a NanoDrop Lite Spectrophotometer (Thermo Fisher Scientific, Inc., Wilmington, DE, United States). The RNA samples were sent to BGI Co., Ltd. (Shenzhen, China) for library preparation and sequencing. Briefly, the polyadenylated mRNA in the samples was enriched using oligo (dT)-attached magnetic beads, and then a fragmentation buffer was used to cut the mRNA into short fragments (approximately 200 bp). cDNA was then synthesized using these cleaved mRNA fragments as templates. The short fragments were then ligated to sequencing adapters, and the products were amplified by PCR. Finally, the cDNA libraries were sequenced on the BGISEQ-500 sequencing platform, and raw data with a read length of 50/100 bp were generated.

### Transcriptome Data Analysis

To obtain clean reads, the raw reads were filtered by trimming the adapter sequences, discarding the reads for which more than 10% of the bases were unknown, and eliminating the reads of which more than 50% of the bases were of low quality. The clean reads were then mapped to the reference genes and genome by Bowtie2 (Kulahoglu and Bräutigam, 2014) and HISTA (Kim et al., 2015), respectively. Gene expression levels were measured by RSEM. The fragments per kilobase of transcript per million mapped reads (FPKM) value was used as a standardized value. Differentially expressed genes (DEGs) were identified according to the default criteria of a fold-change (FC) ≥ 2 and a *Q*-value ≤ 0.001. Gene Ontology (GO) and Kyoto Encyclopedia of Genes and Genomes (KEGG) analyses were carried out to identify the DEGs enriched in GO terms, analyze their metabolic pathways and predict their functions.

### Validation of Differentially Expressed Genes *via* Quantitative Real-Time Polymerase Chain Reaction

To verify the accuracy of the transcriptome data, eight DEGs were randomly selected for quantitative real-time polymerase chain reaction (qPCR), using RNA from biological samples employed for the sequencing analysis (Cervantes-Gámez et al., 2016; Dong et al., 2018). The housekeeping gene *TUBB* was used as a reference gene. Information about the primers used is listed in Supplementary Table 1. qPCR was carried out on a CFX96 Touch™ Real-time PCR Detection System (Bio-Rad Laboratories, Redmond, WA, United States). Each qPCR mixture (total volume of 20 μL) consisted of 1.6 μL of cDNA, 0.8 μL of each forward and reverse primer, 10 μL of 2X SYBR Premix Ex Taq II, and 6.8 μL of ddH_2_O. The PCR program was as follows: denaturation at 95°C for 30 s, followed by 40 cycles at 95°C for 5 s and 60°C for 30 s. Three biological replicates were included for all reactions.

## Results

### Generation of the *lux*AB-Labeled Strain WS32-L

A pTR102 plasmid harboring the *lux*AB gene was transformed into strain WS32 by electrotransformation. A total of 40 transformants were isolated, evaluated and selected. All of these transformants were resistant to tetracycline and kanamycin, and fluoresced in the dark when 10% capraldehyde was added. One of the transformants, namely WS32-L, was selected randomly for further experimentation (Supplementary Figure 1). The 16S rRNA gene sequences of strains WS32 and WS32-L were identical. These results suggest that the *lux*AB gene was successfully transformed into the WS32 strain. The standard growth curves (in LB media) and plant growth-promoting traits, such as phosphate solubilization and siderophore production, were not different between the WS32 and WS32-L strains. The WS32-L colonies retained their fluorescent activity even after 10 generations of continuous culture, indicating that strain WS32-L was genetically stable.

### Colonization Distribution of WS32-L in the Wheat Rhizosphere

To determine the temporal and spatial characteristics of colonization, the labeled strain WS32-L harboring the luciferase *lux*AB gene was inoculated onto the surfaces of germinated wheat seeds. The WS32-L strain was present only on the surface of a small root segment (approximately 0.2–0.3 cm in length) when the wheat seeds were planted. On the 6th day of wheat growth, WS32-L reached 8 cm below the wheat roots, indicating that the strain WS32-L could survive and expand into new zones as the wheat roots grew. The colonization densities of the labeled strain WS32-L on days 12 and 36 were decreased compared with those on day 6. On day 36 of wheat growth, the cell density at depths >8 cm was 1.3 × 10^6^ CFU⋅g^–1^, which was an order of magnitude greater than that at 0–8 cm (Table 1). The cell density of the root segment at 0–8 cm decreased, while that at depths >8 cm remained stable, which indicated that strain WS32-L could move to young roots and colonize new spaces on wheat roots. Taken together, these results indicate that strain WS32-L could survive in the wheat rhizosphere stably and successfully.

**TABLE 1:** Colonization distribution of WS32-L in the wheat rhizosphere (CFU⋅g^–1^).

Time (days)	Root depth (cm)				
	0–2	2–4	4–6	6–8	>8
6	6.1 × 10^9^ ± 5.3 × 10^8^	7.9 × 10^8^ ± 3.6 × 10^7^	7.9 × 10^6^ ± 2.4 × 10^5^	7.1 × 10^6^ ± 2.0 × 10^5^	6.4 × 10^6^ ± 4.1 × 10^5^
12	2.7 × 10^8^ ± 1.6 × 10^7^	5.8 × 10^7^ ± 2.2 × 10^6^	3.6 × 10^6^ ± 1.1 × 10^5^	2.4 × 10^6^ ± 1.3 × 10^5^	1.0 × 10^6^ ± 1.2 × 10^5^
36	3.1 × 10^5^ ± 1.4 × 10^4^	3.3 × 10^4^ ± 2.1 × 10^3^	1.7 × 10^5^ ± 1.2 × 10^4^	1.8 × 10^5^ ± 1.1 × 10^4^	1.3 × 10^6^ ± 2.2 × 10^5^

### Effects of the Growth-Promoting and Phosphate-Solubilizing Strain WS32

To determine the effects of strain WS32 on wheat growth, the seedling fresh and dry weight as well as root fresh and dry weight of 25-day-old wheat plants grown in rhizoboxes were measured (Table 2). The seedling fresh and dry weights were 52.75 and 42.86% higher (*p* < 0.05), respectively, and the root fresh and dry weights were increased by 27.69 and 16.67% (*p* < 0.05), respectively, in the inoculated plants compared with the uninoculated control plants. These results suggest that WS32 promoted the dry matter accumulation of wheat seedlings.

**TABLE 2:** Effects of WS32 on the growth of wheat seedlings collected on day 25 in rhizobox experiments.

Sample	Seedling fresh weight (g)	Seedling dry weight (g)	Root fresh weight (g)	Root dry weight (g)
CK	0.91 ± 0.05	0.14 ± 0.01	0.65 ± 0.12	0.06 ± 0.01
WS32	1.39 ± 0.03*	0.20 ± 0.04*	0.83 ± 0.11*	0.07 ± 0.01*
Percentage increase (%)	52.75	42.86	27.69	16.67

*Indicates a significant difference at p < 0.05. CK: uninoculated wheat seedling. WS32: wheat seedling inoculated with WS32.

**FIGURE 1:**
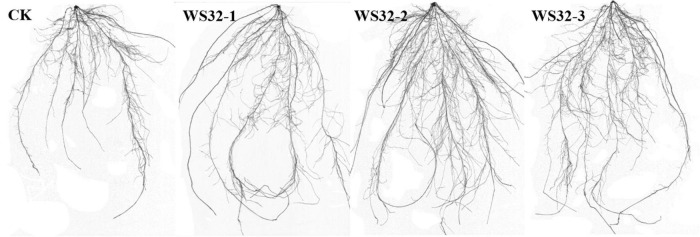
Root morphology of wheat seedlings after 25 days of growth. CK: root system of an uninoculated wheat plant. WS32-1, WS32-2, and WS32-3: root systems of wheat plants inoculated with WS32.

Tracings of the scanned roots of wheat seedlings grown for 25 days in rhizoboxes are shown in Figure 1. The wheat plants inoculated with WS32 had more lateral roots and larger root systems than the uninoculated plants. The data of the parameters related to the root development of wheat are shown in Table 3. The RSA and the numbers of root tips of the inoculated plants were 44.21 and 40.71% (*p* < 0.05) greater, respectively, than those of the uninoculated plants. Strain WS32 promoted the development of the root system of wheat plants. Larger root systems make it easier for wheat plants to absorb nutrients from the soil for growth.

**TABLE 3:** Effects of strain WS32 on wheat root growth collected on day 25 in rhizobox experiments.

Sample	TRL (cm)	RSA (cm^2^)	RAD (mm)	RV (cm^3^)	Tips	Forks
CK	851.99 ± 154.75	91.64 ± 16.84	0.33 ± 0.02	0.79 ± 0.19	2185.33 ± 385.21	5079.33 ± 1538.69
WS32	1229.58 ± 160.54	132.15 ± 18.50*	0.34 ± 0.01	1.13 ± 0.14	3075.00 ± 345.58*	7749.67 ± 1086.98
Percentage increase (%)	44.32	44.21	3.03	43.04	40.71	52.57

*Indicates significant difference at p < 0.05. CK: uninoculated wheat seedling; WS32: wheat seedling inoculated with WS32. TRL, total root length; RSA, root surface area; RAD, root average diameter; RV, root volume.

The phosphorus content of 25-day-old dried leaves was measured, and compared with that of the uninoculated wheat plants (1.72 mg⋅g^–1^), the phosphorus content of wheat plants increased by 49.42% (2.57 mg⋅g^–1^) (*p* < 0.01), suggesting that the WS32 strain can significantly promote phosphorus accumulation in wheat plants.

To explore the phosphate solubilization capacity of strain WS32, the available phosphorus content of the soil after 25 days of wheat growth was also measured. The available phosphorus content of the soil after 25 days of inoculated wheat growth was 18.37 mg⋅kg^–1^, which was not only greater than that of uninoculated wheat seedlings (14.68 mg⋅kg^–1^) (*p* < 0.05) but also higher than that of the soil before planting (15.37 mg⋅kg^–1^). These results indicate that strain WS32 can convert insoluble phosphorus in the soil to soluble phosphorus for wheat absorption and utilization.

Phosphate-solubilizing *Pseudomonas* sp. WS32 has an excellent phosphate solubilization ability and can provide ample amounts of phosphorus nutrients for wheat plant growth and root development. Therefore, strain WS32 is valuable for increasing wheat yields and may be an ideal fertilizer-producing microbial strain for use in sustainable wheat production.

### Quality Analysis of Wheat Root Transcriptome Sequencing Data

To investigate the molecular mechanism of the wheat response to the WS32 strain at the genetic level, transcriptome analysis was performed. A total of six cDNA libraries were constructed. The RNA-seq analysis generated 72.22 Mb of raw reads per biological replicate. After removing low-quality and short sequences, approximately 66 Mb of clean reads were obtained, with Q20 and Q30 percentages of approximately 97.54 and 89.54%, respectively, on average (Supplementary Table 2). Then, the clean reads obtained were mapped to the whole-genome sequence of wheat using HISAT. In the sequenced reads, 91.05–91.35% of the clean reads were well aligned with the wheat reference genome, and 67% on average could be accurately mapped to a specific location within the wheat reference genome sequence. These results indicate that the sequencing was of good quality and contained sufficient information for gene expression analysis.

### Gene Ontology and Kyoto Encyclopedia of Genes and Genomes Enrichment Analysis of Differentially Expressed Genes

**FIGURE 2:**
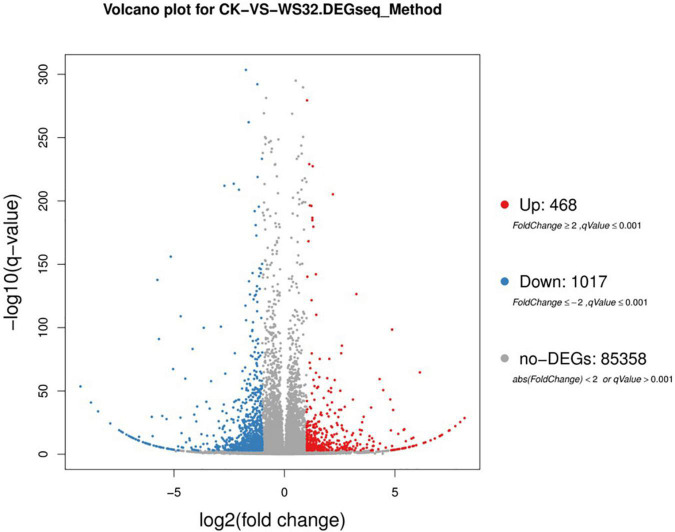
Volcano plot of DEGs in wheat roots inoculated with strain WS32 compared to the controls. The *x*-axis represents log2-transformed FC, and the *y*-axis represents the log10-transformed significance. The red dots represent upregulated genes, the blue dots represent downregulated genes, and the gray dots represent no gene expression.

**FIGURE 3:**
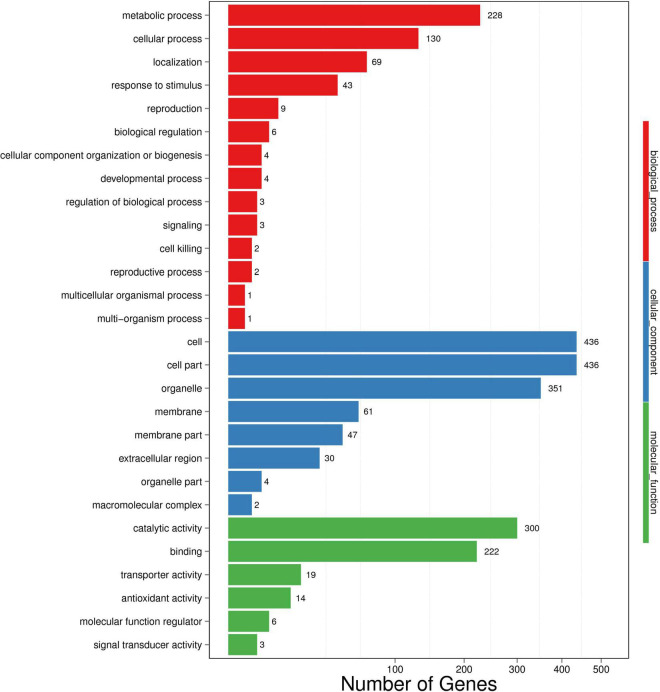
Gene Ontology (GO) enrichment of DEGs identified in wheat. The *x*-axis represents the number of DEGs. The *y*-axis represents the GO terms. Functional annotations of assembled cDNA sequences were based on GO term categorization. Transcripts of the DEGs were further classified into 28 functional subcategories, providing an overview of the ontology content.

Differentially expressed genes (*p* < 0.001 and | log2(FC)| ≥ 1) identified by comparing gene expression levels in wheat roots under inoculated and uninoculated conditions were compared by DESeq2 software. A total of 1485 genes were differentially expressed in the samples of wheat roots inoculated with strain WS32, with 468 upregulated transcripts and 1017 downregulated transcripts (Figure 2). Then, GO functional analysis was performed to classify the functions of DEGs. The GO terms were divided into three categories: those associated with biological processes, cellular components, and molecular functions. The results of the GO functional enrichment are shown in Figure 3. For biological processes, “metabolic process” (228 DEGs), “cellular process” (130 DEGs), and “localization” (69 DEGs) were the three most enriched categories. For the cellular component category, the subcategories with the greatest enrichment and number of DEGs were “cell” (436 DEGs), “cell part” (436 DEGs), and “organelle” (351 DEGs). For the molecular function category, “catalytic activity” (300 DEGs) was the most enriched subcategory, followed by “binding” (222 DEGs).

**FIGURE 4:**
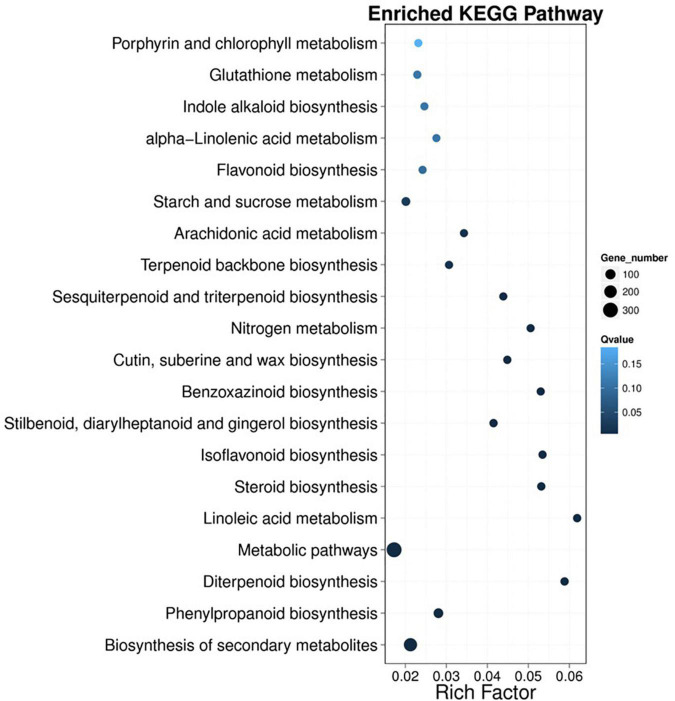
Kyoto Encyclopedia of Genes and Genomes (KEGG) enrichment analysis of DEGs identified in wheat. The *x*-axis represents the enrichment factor. The *y*-axis represents the pathway name. The richness factor refers to the value of the enrichment factor, which is the ratio of the number of DEGs to the total number of DEGs. The larger the value is, the more significant the enrichment.

Kyoto Encyclopedia of Genes and Genomes analyses were performed to better understand the metabolic pathways of the DEGs. A total of 1040 DEGs were annotated and mapped to five main categories that included 19 KEGG pathways. As shown in Figure 4, the greatest number of unigenes was 306, which occurred in “metabolic pathways” (ko0110), followed by 231 unigenes in “biosynthesis of secondary metabolites” (ko01110) and 80 unigenes in “phenylpropanoid biosynthesis” (Ko00940).

### Functional Categories of Differentially Expressed Genes

Most of the transcriptional changes occurred in the functional categories of “phosphorus and other nutrient transport,” “hormone metabolism and organic acid secretion,” “flavonoid signal recognition,” “membrane transport,” and “transcription factor (TF) regulation.” The results are presented in Supplementary Table 3.

#### Phosphorus and Other Nutrient Transport

The “phosphorus and other nutrient transport” category includes eight genes that were differentially expressed in the roots of wheat plants inoculated by WS32 or not, 2 of which were related to phosphorus transport and 6 of which were related to nitrate transport. In this study, it was determined that the expression of the phosphate transporter gene *TaPHT1;4* and the *SPX* gene, which have been reported to be significantly enhanced under phosphorus deficiency and reduced under high-phosphorus conditions, were downregulated by the activity of the phosphate-solubilizing strain WS32 (TRIAE_CS42_4AL_TGACv1_289415_AA0970660, TRIAE_CS42_6BL_TGACv1_500863_AA1610920), indicating that WS32 may solubilize insoluble phosphorus in the soil to provide a rich phosphorus environment for wheat plants.

Kyoto Encyclopedia of Genes and Genomes metabolism showed that, in addition to those of phosphate transporters, the expression levels of six high-affinity nitrate transporters in wheat roots increased after the application of the phosphate-solubilizing strain WS32. Moreover, strain WS32 could promote wheat growth by inducing the expression of high-affinity nitrate transporter genes in wheat roots.

#### Hormone Metabolism and Organic Acid Secretion

The “hormone metabolism and organic acid secretion” category includes 20 genes that were identified as WS32 responsive, namely, five upregulated DEGs and 15 downregulated DEGs. Four subcategories were mainly represented: *MYC2*-related genes regulating the jasmonic acid signaling pathway (TRIAE_CS42_5AS_TGACv1_392955_AA1266770, TRIAE_CS42_6BL_TGACv1_501219_AA1614960) and genes involved in malic acid biosynthesis (TRIAE_CS42_3DL_TGACv1_249039_AA0835670) were upregulated, while pyrophosphate synthase genes involved in the gibberellin biosynthesis pathway and genes encoding the auxin-induced protein 5NG4 in wheat roots were downregulated. These results indicate that the WS32 strain could regulate the growth and development of wheat by regulating the production of wheat hormones and the secretion of organic acids.

#### Flavonoid Signal Recognition

As shown in Supplementary Table 2, 18 genes related to flavonoid signal recognition were differentially expressed in wheat roots after inoculation with strain WS32. Six genes encoding the O-methyltransferase ZRP4 were upregulated. Two genes homologous to the flavonoid O-methyltransferase gene were upregulated. Four DEGs were involved in regulating flavonol synthase, three of which were downregulated and one of which was upregulated. After inoculation with the WS32 strain, the expression of the isoflavone 2′-hydroxylase gene significantly increased.

#### Membrane Transport

The category “membrane transport” includes 12 genes that present different expression levels between inoculated and uninoculated wheat roots. Seven of these membrane transport genes were annotated as ABC transporters, and five of them were annotated as phospholipases. All 12 genes were downregulated after inoculation with WS32.

#### Transcription Factor Regulation

Transcription factors, which can specifically bind to corresponding *cis*-elements and induce downstream gene expression when activated, play a variety of important roles in plant development and responses to abiotic stress (Wang et al., 2021). In this study, the expression levels of 25 TFs in wheat roots changed significantly after inoculation with WS32. The five main groups (and their associated number of DEGs) in this subcategory were as follows: MYB TFs, with nine DEGs, all of which were downregulated; AP2/ERF TFs, with five downregulated DEGs and one upregulated DEG; MADS-box TFs, which included two downregulated DEGs and two upregulated DEGs; basic helix-loop-helix (bHLH) TFs, which included two downregulated DEGs and one upregulated DEG; and WRKY TFs, with three downregulated genes. MYB and WRKY TFs are mainly involved in the response of plants to temperature stress and are induced under stress conditions (Li et al., 2020; Wang et al., 2021). The downregulated expression of MYB and WRKY TFs indicated that the phosphate-solubilizing strain WS32 may be capable of improving the disease resistance and stress resistance of wheat. High concentrations of ethylene inhibit plant root growth, and ethylene response factors (AP2s/ERFs) could modulate ethylene biosynthesis through a feedback-driven process (Zhang et al., 2019). In this study, AP2/ERF TFs of wheat were downregulated after the roots were inoculated with strain WS32, indicating that strain WS32 could inhibit the expression of the ethylene AP2/ERF TF to promote wheat root growth and enhance root activity. MADS-box TFs control developmental processes of plant roots (Zhang Z. B. et al., 2012). By increasing their own transcription under low-phosphorus conditions, bHLH TFs can enhance plant tolerance to phosphorus deficiency (Li et al., 2014). The expression levels of bHLH TFs changed in response to inoculation with WS32, indicating that strain WS32 may increase the phosphorus content in wheat rhizosphere soil and restore the expression of bHLH, albeit to a lower level than normal.

### Validation of RNA Sequencing Data

**FIGURE 5:**
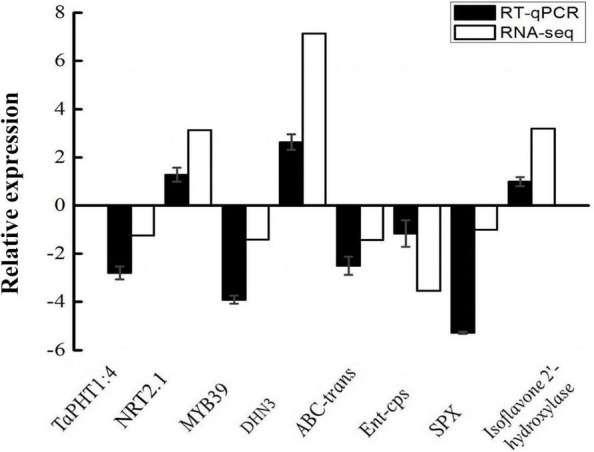
Validation of RNA-seq expression profiles *via* qPCR.

To validate the RNA-seq data, eight DEGs were selected randomly for qPCR analysis, wherein the *TUBB* gene was used as a reference gene. One hundred percent of the genes examined by qPCR displayed the same expression trends as those observed in the RNA-seq experiment (Figure 5). Thus, the qPCR and RNA-seq results were highly consistent, indicating the accuracy and reliability of the transcriptome sequencing results.

## Discussion

### Rhizosphere Colonization, Phosphate-Solubilizing Ability and Growth-Promoting Effects of Strain WS32 on Wheat

Beneficial interactions between plants and microorganisms play important roles in plant growth and development (Jeffries et al., 2003). Molecules exuded by plant roots can be regarded as nutrient components that could support the growth of microorganisms in the rhizosphere (Zaidi et al., 2009). Moreover, PGPR promote the growth and development of their host plants through several mechanisms, including solubilizing phosphorus and potassium, fixing N_2_ (Zaidi et al., 2009; Ahemad and Khan, 2012), producing siderophores, producing phytohormones and secreting antibiotics (Artursson et al., 2006). Many studies have reported that PGPR promote plant growth. Cao et al. (2020) reported that inoculation of tobacco with *Pseudomonas* sp. TK35-L can significantly improve tobacco root growth and dry matter accumulation. Hassan and Bano (2015) isolated a *Pseudomonas moraviensis* strain from the rhizosphere of halophytic weed, and the isolate was found to enhance growth and physiology of wheat plants. Several studies have focused on the colonization level of PGPR (Loján et al., 2017; Santoyo et al., 2021). Santoyo et al. (2021) studied the different colonization strategies of PGPR in the rhizosphere of host plants, and Loján et al. (2017) evaluated the impact of seven different PGPR on root colonization and the life cycle of *Rhizophagus irregularis* MUCL 41833. However, these studies focused on the efficacy of PGPR to play a growth-promoting role and the colonization level of PGPR but did not assess the relationship between the ability of PGPR to colonize the rhizosphere and to promote root growth development. Xu et al. (2018) verified that rhizosphere colonization by PGPR is a pre-requisite for effective promotion of plant growth. Liu et al. (2019) indicated that root colonization is critical for PGPR to promote plant growth. However, effective colonization of PGPR means not only that the strain can survive within the rhizosphere for long periods but also that the strain can expand into new zones following plant root extension (Cao et al., 2020). The ability of PGPR to recognize their host plants and to colonize the rhizosphere depends on the combination of lectins secreted by plants and polysaccharides secreted by the PGPR (Komath et al., 2006). An affinity for wheat lectins is a pre-requisite for effective wheat rhizosphere colonization by PGPR. Based on this information, the PGPR strain *Pseudomonas* sp. WS32, which as an affinity for wheat lectins and presents great plant growth-promoting traits, was isolated from the wheat rhizosphere through a new technique that we established involving the use wheat germ agglutinin (Zhang J. et al., 2012). In the present study, we focused on WS32 colonization of the wheat rhizosphere and colonization-induced changes in both wheat development and genes expression in wheat roots. The *lux*AB gene was introduced into strain WS32 by electrotransformation to track target strains in the soil, and then a labeled strain (WS32-L) was generated and used as a research object. WS32-L maintained a high density at wheat root depths >8 cm following root growth, indicating that the strain WS32-L had an affinity for wheat and could expand in new spaces with the growth and extension of roots. On the 36th day of wheat growth, the colonization density of WS32-L decreased within the 0–8 cm root depth but remained high at the >8 cm root depth. This may have occurred because the 0–8 cm root segments eventually became old roots, for which the amount of produced root exudates available for microorganisms decreased, leading to a gradual reduction in the number of strain WS32-L bacteria on the root surface; thus, the strain WS32-L bacteria migrated to and settled on young roots as they grew. Liu et al. (2019) reported that *Pseudomonas* sp. P34-L colonized the wheat rhizosphere with higher density and activity in new roots compared with older roots, which coincidently with this study. Our findings indicated that strain WS32-L bacteria could not only colonize the wheat rhizosphere for long periods but also migrate and settle to deep root depths with the extension of wheat roots. Moreover, strain WS32-L increased seedling fresh weight, seedling dry weight, and root fresh and dry weights of wheat plants; increased the RSA and the numbers of root tips and forks; and enhanced the phosphorus accumulation both in rhizosphere soil and in the leaves of wheat. Increased phosphorus accumulation in wheat plants promoted the development of the wheat roots and then played a positive role in wheat growth. Moreover, strain WS32-L could survive in the wheat rhizosphere for a long time, indicating that this strain can colonize and is adaptable to the wheat rhizosphere. Thus, this strain can survive and persist in the wheat rhizosphere and continuously produce beneficial substances for the development of wheat root systems to promote the whole growth and development of wheat.

### WS32 Rhizosphere Colonization-Induced Expression Changes in Wheat Roots

At the molecular level, plant growth and development are regulated by various genes. In this study, using RNA-seq technology we explored the molecular mechanism underlying the growth-promoting effects of the PGPR strain WS32. RNA-seq is an efficient method that can generate an abundance of transcript data, and compared with conventional methods, this method is more sensitive, is more repeatable, and has a wider dynamic range. In this study, we used RNA-seq technology and identified 1485 genes that were differentially expressed in the roots of wheat plants inoculated or not with WS32, some of which were associated with phosphorus and other nutrient transport. Phosphorus (P) and nitrogen (N) are indispensable nutrients and are required for normal plant growth and development. Here, we focused on two downregulated DEGs and six upregulated DEGs, which included the phosphate transporter genes *TaPHT1;4* and *SPX* and nitrate transporter genes, in the “phosphorus and other nutrient transport” subcategory, whose members are annotated as transporters. The *TaPHT1;4* gene and *SPX* gene were found to be expressed under low-phosphorus conditions (Jung et al., 2017). Liu et al. (2013) found that *TaPHT1;4* transcripts were specifically detected in wheat roots and that *TaPHT1;4* was upregulated under P deficiency but downregulated under adequate phosphorus levels. Kumar et al. (2019) utilized qPCR to verify the expression level of the *SPX* gene in the wheat genome under different phosphorus conditions and found that the *SPX* gene was upregulated under phosphorus-deficient conditions and downregulated under high-phosphorus conditions, indicating that the *SPX* family is involved in the expression of phosphorus signaling pathway component genes in wheat. The results of this study showed that the expression levels of the phosphorus transporter genes *TaPHT1:4* and *SPX* were significantly downregulated after inoculation with WS32, indicating that the WS32 strain could increase the phosphorus content in the wheat rhizosphere. The expression of *NRT2.1*, which is a member of the *NRT2* family and is involved in nitrate transport, was induced in this study. Nitrate (NO_3_^–^) is one of the available biological forms of N and a major N source for higher plants (Martin and Marschner, 1988). The most substantial effect of nitrate is stimulation of the formation of lateral roots, which can increase the uptake of nutrients and water by wheat plants. At present, the expression of orthologous *NRT2* family genes has been reported in Arabidopsis, maize and rice, and it is believed that *NRT2* family genes are heterologously expressed in rhizobia and induced by exposure to certain nitrate concentrations. High expression of *NRT2* can promote the uptake of nitrate by plants (Orsel et al., 2002; Garnett et al., 2013). WS32 could therefore promote the uptake of nitrate by wheat roots and stimulate the development of lateral roots.

Plant hormones and organic acids also play important roles in the growth and development of wheat. In the present study, 20 genes were identified as being WS32 responsive, including *MYC2* and auxin-inducible protein 5NG4, which were identified as regulating both hormone synthesis and organic acid secretion. *MYC2* has been determined to be a master regulator of jasmonate (JA)-mediated responses as well as crosstalk among different signaling pathways (Guo et al., 2012). JAs constitute a group of plant growth regulators and signaling molecules that have pivotal roles in plant root growth, fertility, and responses to abiotic and biotic stresses (He et al., 2002). Meng et al. (2005) found that inoculation with *Mitsuaria* sp. ADR 17 could increase the content of JAs in plants. In the present study, the expression level of *MYC2* was upregulated after inoculation with strain WS32, indicating that strain WS32 could regulate the jasmonic acid synthesis pathway in wheat roots and thus promote wheat growth. Moreover, we found that the expression of the auxin-induced protein 5NG4 gene was downregulated. The auxin-induced protein 5NG4 gene was detected in loblolly pine and was found to be involved in the regulation of root growth, but this gene was downregulated in shoots in which vigorous metabolic activity was occurring (Busov et al., 2004). These results indicate that the PGPR strain WS32 could enhance the root activity of wheat plants in the early growth and development stages.

Rhizosphere colonization is a pre-requisite for PGPR to promote growth. The colonization of PGPR involves a complex process of signal exchange and mutual recognition. Flavonoids are the most extensive secondary metabolites in plants and play important roles as signaling molecules in plant-rhizobacteria interactions (Cesari et al., 2020). Isoflavonoids represent a subclass of flavonoids and signaling molecules secreted by plants and are believed to play an important role in plant–microbe interactions (Rolfe, 1988). Isoflavonoids are released in a host-specific manner but formed through the same flavonoid biosynthetic pathway (Badri et al., 2009; Barnes, 2010). Isoflavone 2′-hydroxylase is involved in the synthesis of isoflavone derivatives to improve the ability of wheat to resist stress (Akashi et al., 1998). O-methyltransferase methylation of flavonoid substances produces flavonoid methyl derivatives, which, as signaling molecules in flavonoid biosynthesis, play important roles in antiviral and plant-bacterium interactions (Liu et al., 2020). Isoflavone 2′-hydroxylase and O-methyltransferase ZRP4, as well as flavonoid O-methyltransferase, which are involved in the synthesis of flavonoids, were upregulated in the inoculated wheat roots, indicating that strain WS32 can promote the secretion of flavonoids in wheat. Moreover, long-term adsorption of PGPR strain WS32, which has an affinity for wheat, onto wheat roots can be promoted through recognition and communication with flavonoids secreted by the wheat plants during bacterium-plant interactions.

Interactions between plants and microorganisms involve complex signal regulation and transmission, which depend on the regulation of a series of intracellular transporters and synthesis of biofilms. Twelve membrane transporters, namely, seven ABC transporters and five phospholipases, were identified as being differentially expressed in this study. The ABC transporter family is a class of superfamily transmembrane proteins that are widely present in organisms and play important roles in secondary metabolite transport and accumulation in plants (Badri et al., 2009; Yan et al., 2017; Qu et al., 2020). Suchan et al. (2020) found that ABC transporter-related genes were upregulated according to the transcriptome of soybean inoculated with *Delftia acidovorans* RAY209. The results of the present study showed that the expression levels of ABC transporter genes and phospholipase genes related to membrane synthesis were differentially expressed in wheat roots, which may be caused by the strain WS32 bacteria inducing ABC transporters to release energy through the hydrolysis of ATP and transport of nutrients across the membrane into wheat cells, thus inducing changes in membrane transporter activity and synthesis-related genes.

Specific TFs are likely to play important roles in the regulation of the WS32-induced responses in wheat roots. The results of this study showed that members of TF gene families were differentially expressed in response to the WS32 strain, and most of these genes belonged to the MYB, MADS-box, bHLH, WRKY, and AP2/ERF families. MYB and WRKY TFs are mainly involved in the response of plants to temperature stress (Li et al., 2020; Wang et al., 2021). High concentrations of ethylene inhibit plant root growth, and ethylene response factors (AP2s/ERFs) could modulate ethylene biosynthesis through a feedback-driven process (Zhang et al., 2019). MADS-box TFs control developmental processes of plant roots (Zhang Z. B. et al., 2012). By increasing their own transcription under low-phosphorus conditions, bHLH TFs can enhance plant tolerance to phosphorus deficiency (Li et al., 2014). After inoculation with the phosphate-solubilizing bacterial strain WS32, several anti-stress-related TF genes were downregulated. The differential expression levels of TFs could lead to complex changes in the development and metabolism of wheat roots. Cervantes-Gámez et al. (2016) showed that arbuscular mycorrhizae were involved in the regulation of defense-related WRKY TFs in interactions between arbuscular mycorrhizal fungi and *Solanum*. Meng et al. (2005) found that *Mitsuaria* sp. ADR17 produced extracellular polysaccharides that significantly alleviated plant stress. Our results showed that the expression of several anti-stress–related TF genes was downregulated, which may have occurred because, by secreting corresponding extracellular polysaccharides, strain WS32 improved the stress resistance ability of wheat. MADS-box TF genes were upregulated; however, the structure and complexation of these TFs have not been well characterized, and their regulatory mechanisms need to be further verified.

## Conclusion

Phosphate-solubilizing *Pseudomonas* sp. strain WS32 could survive in the wheat rhizosphere for long periods and colonize new zones following wheat root extension, exhibiting significant increases in seedling growth, root development, and phosphorus accumulation in the wheat leaves. A total of 1485 genes in wheat roots were differentially expressed between the inoculated conditions and the uninoculated conditions. Most of the transcriptional changes occurred for genes annotated to the following functional categories: “phosphorus and other nutrient transport,” “hormone metabolism and organic acid secretion,” “flavonoid signal recognition,” “membrane transport,” and “TF regulation.” These findings lay a foundation for future studies on the molecular mechanism underlying the plant growth-promoting activities of PGPR, and provide theoretical basis and experimental data for crop rhizosphere regulation.

## Data Availability Statement

The datasets presented in this study can be found in online repositories. The names of the repository/repositories and accession number(s) can be found below: https://www.ncbi.nlm.nih.gov/, PRJNA827998.

## Author Contributions

KO and XH carried out the experiments and participated in the data analysis and manuscript writing. KC, WZ, XJ, WA, and YD carried out the experiments and participated in the data analysis. YC acquired the funding, designed the study, analyzed the data, and wrote the manuscript. All authors have read and approved the manuscript.

## Conflict of Interest

The authors declare that the research was conducted in the absence of any commercial or financial relationships that could be construed as a potential conflict of interest.

## Publisher’s Note

All claims expressed in this article are solely those of the authors and do not necessarily represent those of their affiliated organizations, or those of the publisher, the editors and the reviewers. Any product that may be evaluated in this article, or claim that may be made by its manufacturer, is not guaranteed or endorsed by the publisher.

## Supplementary Material

Supplementary Figure 1 Fluorescence emitted by WS32-L colonies.
