# Supplementary figures and images for: Phosphate-Solubilizing Pseudomonas sp. Strain WS32 Rhizosphere Colonization-Induced Expression Changes in Wheat Roots
Source: Front Microbiol. 2022 Jun 30;13:927889. doi: 10.3389/fmicb.2022.927889 (PMC9279123; doi:10.3389/fmicb.2022.927889)

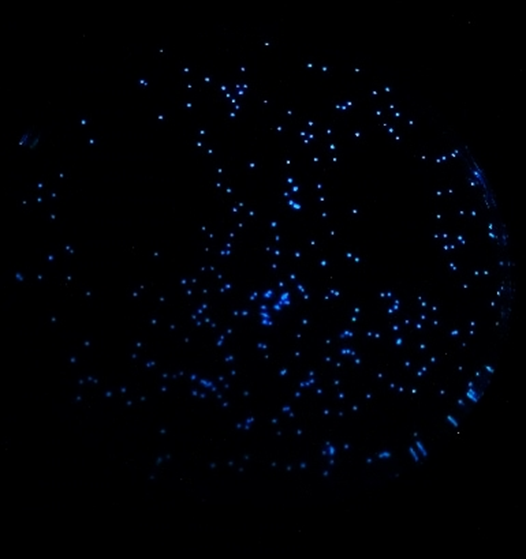

Supplement: Supplementary Figure 1 — Fluorescence emitted by WS32-L colonies. [file Image_1.tif]
