# Supplementary material for: Phosphate-Solubilizing Pseudomonas sp. Strain WS32 Rhizosphere Colonization-Induced Expression Changes in Wheat Roots
Source: Front Microbiol. 2022 Jun 30;13:927889. doi: 10.3389/fmicb.2022.927889 (PMC9279123; doi:10.3389/fmicb.2022.927889)
Supplement: Supplementary file 2 [file Table_1.docx]

**Supplementary material**

**Table S1.** Primers used in RT-qPCR

| Gene ID | Forward (5’-3’) | Reverse(5’- 3’) |
| --- | --- | --- |
| *TUBB* | CAAGGAGGTGGACGAGCAGATG | GACTTGACGTTGTTGGGGATCCA |
| *MYB39* | CAACACTGCGATGAGGGAGAG | CTGGGAAACGGACTACTGACG |
| *DHN3* | ATGAGGGACGAGCACCAGAC | CTTCTTCCTCCTCCCGCC |
| *ent-CPS* | ATGGATACACCGTCTCACCGA | CCTGGGCTTCCCTTTCTCTAA |
| *NRT2.1* | TTCGTGCCAGGAATGATGC | TAGCCGTAGAGGAGGACAAAGAT |
| *Isoflavone2’-hydroxylase* | GCATCCACCAACACCACTTCT | CGTCGCCTGTCCATACCAA |
| *ABC-trans* | AGAGCGAGCACACCAGCCT | TCTTCCTCTGCTTGACGCTGT |
| *SPX* | AACACATACATCATCGTGCCAAC | ATAATCACACCGCAAACAGTCG |
| *TaPHT1:4* | AGAAACTCACGCACAGCACAAC | GCACCAAGGCTCACCG |
