# Supplementary material for: Phosphate-Solubilizing Pseudomonas sp. Strain WS32 Rhizosphere Colonization-Induced Expression Changes in Wheat Roots
Source: Front Microbiol. 2022 Jun 30;13:927889. doi: 10.3389/fmicb.2022.927889 (PMC9279123; doi:10.3389/fmicb.2022.927889)
Supplement: Supplementary file 3 [file Table_2.docx]

**Supplementary material**

**Table S2.** Quality analysis of raw reads

| Sample | Total Raw Reads (Mb) | Total Clean Reads (Mb) | Total Clean Bases (Gb) | Clean Reads Q20 (%) | Clean Reads Q30 (%) |
| --- | --- | --- | --- | --- | --- |
| CK-1 | 72.22 | 66.50 | 6.65 | 97.49 | 89.42 |
| CK-2 | 72.22 | 66.93 | 6.69 | 97.59 | 89.65 |
| CK-3 | 72.22 | 66.98 | 6.70 | 97.62 | 89.82 |
| WS32-1 | 72.22 | 66.99 | 6.70 | 97.52 | 89.45 |
| WS32-2 | 72.22 | 66.48 | 6.65 | 97.52 | 89.51 |
| WS32-3 | 72.22 | 66.65 | 6.67 | 97.50 | 89.41 |
