# Supplementary material for: Phosphate-Solubilizing Pseudomonas sp. Strain WS32 Rhizosphere Colonization-Induced Expression Changes in Wheat Roots
Source: Front Microbiol. 2022 Jun 30;13:927889. doi: 10.3389/fmicb.2022.927889 (PMC9279123; doi:10.3389/fmicb.2022.927889)
Supplement: Supplementary file 4 [file Table_3.docx]

**Supplementary material**

**Table S3.** Functional classification of the differentially expressed genes

| Gene ID | Nr Description | log2Ratio | FDR(q-value) |
| --- | --- | --- | --- |
| **Phosphorus and other nutrient transport** |  |  |  |
| TRIAE_CS42_4AL_TGACv1_289415_AA0970660 | Phosphate transporter protein *TaPHT1;4* | -1.2139 | 1.36e-219 |
| TRIAE_CS42_6BL_TGACv1_500863_AA1610920 | SPX domain-containing membrane protein Os02g45520 | -1.0574 | 6.51e-28 |
| TRIAE_CS42_6BS_TGACv1_513763_AA1648890 | Nitrate transporter | 2.2732 | 2.55e-59 |
| TRIAE_CS42_6AS_TGACv1_485608_AA1548630 | High-affinity nitrate transporter 2.1 | 2.2522 | 2.90e-16 |
| TRIAE_CS42_6AS_TGACv1_485608_AA1548640 | High affinity nitrate transporter2 | 1.8132 | 5.70e-12 |
| TRIAE_CS42_6BS_TGACv1_513763_AA1648870 | High affinity nitrate transporter *TaNRT2.1* | 1.5461 | 7.15e-18 |
| TRIAE_CS42_6DS_TGACv1_542679_AA1727350 | High-affinity nitrate transporter 2.1 | 1.4635 | 1.53e-22 |
| TRIAE_CS42_6BS_TGACv1_513763_AA1648900 | High affinity nitrate transporter | 1.3614 | 1.72e-29 |
| **Hormone metabolism and organic acid secretion** |  |  |  |
| TRIAE_CS42_5AS_TGACv1_392955_AA1266770 | Transcription factor *MYC2* | 1.2945 | 7.63e-11 |
| TRIAE_CS42_1DL_TGACv1_062922_AA0221730 | Auxin -induced protein 5NG4 | 1.6056 | 5.06e-20 |
| TRIAE_CS42_6AL_TGACv1_473168_AA1529180 | Auxin -induced protein 5NG4 | -1.1358 | 4.04e-10 |
| TRIAE_CS42_2DL_TGACv1_158177_AA0511450 | Auxin -induced protein 5NG4 | -1.4947 | 2.78e-09 |
| TRIAE_CS42_5DS_TGACv1_456837_AA1478910 | Auxin -induced protein 5NG4 | -1.1945 | 4.95e-08 |
| TRIAE_CS42_3AS_TGACv1_210508_AA0674210 | Auxin -induced protein 5NG4 | -1.2301 | 6.32e-08 |
| TRIAE_CS42_1AL_TGACv1_003529_AA0050250 | Auxin -induced protein 5NG4 | 1.1202 | 8.30e-08 |
| TRIAE_CS42_6AL_TGACv1_472793_AA1526050 | Auxin -induced protein 5NG4 | -1.9015 | 8.61e-08 |
| TRIAE_CS42_2AL_TGACv1_093314_AA0277100 | Auxin -induced protein 5NG4 | -1.0685 | 3.14e-06 |
| TRIAE_CS42_2DL_TGACv1_161791_AA0560610 | Auxin -induced protein 5NG4 | -1.5417 | 0.0004433 |
| TRIAE_CS42_7BL_TGACv1_577914_AA1885950 | Ent-copalyl diphosphate synthase | -1.1103 | 6.10e-06 |
| TRIAE_CS42_7DL_TGACv1_602609_AA1962550 | Ent-copalyl diphosphate synthase | -1.2438 | 3.55e-14 |
| TRIAE_CS42_2AS_TGACv1_112552_AA0340650 | Ent-copalyl diphosphate synthase | -1.2931 | 0.0001080 |
| TRIAE_CS42_7AL_TGACv1_556210_AA1758330 | Ent-copalyl diphosphate synthase | -1.5651 | 6.53e-11 |
| TRIAE_CS42_3AL_TGACv1_194533_AA0635230 | Ent-copalyl diphosphate synthase | -1.8247 | 7.71e-05 |
| TRIAE_CS42_U_TGACv1_641065_AA2083870 | Ent-copalyl diphosphate synthase | -2.6529 | 1.46e-10 |
| TRIAE_CS42_2BL_TGACv1_129351_AA0379810 | Ent-copalyl diphosphate synthase | -3.5350 | 3.44e-42 |
| TRIAE_CS42_2AS_TGACv1_116313_AA0373780 | Ent-copalyl diphosphate synthase | -3.9481 | 2.20e-38 |
| TRIAE_CS42_3DL_TGACv1_249039_AA0835670 | NADP-dependent malic enzyme | 2.4743 | 8.23e-36 |
| **Flavonoid signal recognition** |  |  |  |
| TRIAE_CS42_3DL_TGACv1_249919_AA0858550 | Isoflavone 2'-hydroxylase | 3.5834 | 2.55e-30 |
| TRIAE_CS42_6BL_TGACv1_505061_AA1629060 | O-methyltransferase ZRP4 | 1.3979 | 6.52e-182 |
| TRIAE_CS42_4DL_TGACv1_345885_AA1154620 | O-methyltransferase ZRP4 | 1.1231 | 1.04e-229 |
| TRIAE_CS42_4DS_TGACv1_362882_AA1182540 | O-methyltransferase ZRP4 | 1.0356 | 7.25e-141 |
| TRIAE_CS42_6BL_TGACv1_508743_AA1629720 | O-methyltransferase ZRP4 | 1.4375 | 7.89e-111 |
| TRIAE_CS42_U_TGACv1_642881_AA2124380 | O-methyltransferase ZRP4 | 1.0252 | 5.66e-17 |
| TRIAE_CS42_7AS_TGACv1_570448_AA1835810 | O-methyltransferase ZRP4 | 2.1280 | 1.06e-15 |
| TRIAE_CS42_6DL_TGACv1_528583_AA1715140 | Anthranilate N-benzoyltransferase protein | -1.4828 | 1.56e-20 |
| TRIAE_CS42_5DL_TGACv1_433883_AA1424460 | Shikimate O-hydroxycinnamoyltransferase | -1.5733 | 6.91e-11 |
| TRIAE_CS42_2BL_TGACv1_129297_AA0377120 | Agmatine coumaroyltransferase-1 | -3.0016 | 3.63e-07 |
| TRIAE_CS42_2DL_TGACv1_161624_AA0559560 | Agmatine coumaroyltransferase-1 | -1.9736 | 8.56e-06 |
| TRIAE_CS42_6BL_TGACv1_499746_AA1590940 | Anthranilate N-benzoyltransferase protein | -1.3376 | 3.54e-05 |
| TRIAE_CS42_7AS_TGACv1_571273_AA1846510 | Protein SRG1 | -4.4528 | 4.01e-10 |
| TRIAE_CS42_5AL_TGACv1_374015_AA1187770 | Flavonol synthase | 1.0371 | 2.58e-06 |
| TRIAE_CS42_7DS_TGACv1_621940_AA2029810 | Protein SRG1 | -5.0890 | 0.00015 |
| TRIAE_CS42_7DS_TGACv1_621705_AA2023800 | Protein SRG1 | -2.2265 | 0.00056 |
| BGI_novel_G004466 | Flavonoid O-methyltransferase-like protein Os11g0303600 | 1.3063 | 2.3e-180 |
| BGI_novel_G006295 | Flavonoid O-methyltransferase-like protein Os11g0303600 | 1.4230 | 8.21e-143 |
| **Membrane transport** |  |  |  |
| TRIAE_CS42_4BL_TGACv1_320780_AA1048570 | ABC transporter G family member 16 | -1.2600 | 5.00e-72 |
| TRIAE_CS42_7DS_TGACv1_621658_AA2022360 | ABC transporter G family member 45-like isoform X1 | -1.2633 | 0.000602 |
| TRIAE_CS42_4DL_TGACv1_343053_AA1128620 | ABC transporter G family member 16 | -1.2646 | 9.05e-73 |
| TRIAE_CS42_4AS_TGACv1_308827_AA1029610 | ABC transporter G family member 16 | -1.4461 | 6.90e-78 |
| TRIAE_CS42_2AL_TGACv1_097112_AA0323000 | ABC transporter G family member 37 | -2.02758 | 1.71e-23 |
| TRIAE_CS42_7BS_TGACv1_592389_AA1937190 | ABC transporter C family member 10 | -2.36713 | 2.98e-06 |
| BGI_novel_G003375 | ABC transporter G family member 53 | -2.63908 | 2.02e-10 |
| TRIAE_CS42_6AS_TGACv1_485375_AA1544430 | Phospholipase D delta | -1.09027 | 0.000552 |
| TRIAE_CS42_U_TGACv1_644136_AA2137450 | Phospholipase D delta | -1.15691 | 7.54e-05 |
| TRIAE_CS42_7AL_TGACv1_556845_AA1771970 | Secretory phospholipase A2 | -1.42607 | 0.000622 |
| TRIAE_CS42_7DL_TGACv1_603979_AA1991900 | Putative phospholipase A2 | -2.00158 | 1.18e-09 |
| TRIAE_CS42_7BL_TGACv1_577295_AA1871440 | Putative phospholipase A2 | -2.47551 | 6.18e-14 |
| **Transcription Factor regulation** |  |  |  |
| TRIAE_CS42_6AL_TGACv1_471197_AA1504490 | Transcription factor MYB39 | -1.40221 | 0.000687 |
| TRIAE_CS42_6BL_TGACv1_499853_AA1593300 | Transcription factor MYB39 | -1.10251 | 1.78e-05 |
| TRIAE_CS42_7DS_TGACv1_623297_AA2051700 | Transcription factor MYB39 | -1.46531 | 0.000552 |
| TRIAE_CS42_6AL_TGACv1_472122_AA1517970 | Transcription factor MYB39 | -1.46368 | 7.54e-05 |
| TRIAE_CS42_6BL_TGACv1_501605_AA1619280 | Transcription factor MYB39 | -1.43126 | 0.000622 |
| TRIAE_CS42_4AL_TGACv1_290671_AA0988150 | Transcription factor MYB39 | -1.31154 | 1.18e-09 |
| TRIAE_CS42_6DL_TGACv1_526829_AA1692860 | Transcription factor MYB39 | -1.15912 | 6.18e-14 |
| TRIAE_CS42_7DS_TGACv1_621471_AA2016630 | Transcription factor MYB39 | -1.6169 | 0.000687 |
| TRIAE_CS42_4AL_TGACv1_290858_AA0990120 | Transcription factor MYB39 | -1.5798 | 1.78e-05 |
| BGI_novel_G003707 | WRKY transcription factor *WRKY62* | -2.9392 | 2.36e-21 |
| TRIAE_CS42_2DS_TGACv1_177727_AA0583270 | Putative WRKY transcription factor 38 | -1.0220 | 5.46e-06 |
| TRIAE_CS42_3AL_TGACv1_196498_AA0660850 | Putative WRKY transcription factor 24 | -1.2939 | 0.000359 |
| TRIAE_CS42_4DL_TGACv1_343669_AA1138030 | Transcription factor *bHLH93* | 1.1799 | 4.55e-05 |
| TRIAE_CS42_6BL_TGACv1_501219_AA1614960 | Transcription factor *bHLH93* | 1.1799 | 4.55e-05 |
| TRIAE_CS42_7DL_TGACv1_603113_AA1976200 | Transcription factor *bHLH144* | -1.0447 | 3.57e-11 |
| TRIAE_CS42_2BS_TGACv1_146126_AA0455980 | Transcription factor *bHLH54* | -3.4420 | 5.76e-05 |
| TRIAE_CS42_5BS_TGACv1_423180_AA1369410 | MADS-box transcription factor TaAGL31 | 3.9149 | 0.000954 |
| TRIAE_CS42_1BS_TGACv1_050417_AA0171830 | MADS-box transcription factor 27 | 1.2817 | 1.35e-07 |
| TRIAE_CS42_7AL_TGACv1_557712_AA1785060 | MADS-box transcription factor 26 | -1.2521 | 1.23e-06 |
| TRIAE_CS42_5AL_TGACv1_374176_AA1192420 | MADS-box transcription factor | -1.62 | 9.09e-07 |
| TRIAE_CS42_5AL_TGACv1_375132_AA1216540 | AP2 domain CBF protein | 1.83131 | 7.61e-06 |
| TRIAE_CS42_3AS_TGACv1_210918_AA0681430 | AP2/ERF and B3 domain-containing protein Os01g0141000 | -1.3286 | 0.000142 |
| TRIAE_CS42_4DL_TGACv1_342392_AA1112370 | AP2-like ethylene-responsive transcription factor | -2.2544 | 0.000434 |
| TRIAE_CS42_5AS_TGACv1_392716_AA1263460 | Ethylene-responsive transcription factor RAP2-11 | -3.0378 | 9.86e-05 |
| TRIAE_CS42_4AS_TGACv1_306883_AA1014550 | Ethylene-responsive transcription factor 1B | -1.9756 | 3.38e-06 |
| TRIAE_CS42_5AL_TGACv1_373962_AA1185480 | Ethylene-responsive transcription factor 1 | -2.4388 | 5.21e-09 |
